# Supplementary figures and images for: Unveiling hidden rocky reefs of the Mexican Atlantic coast: Topographic characterization and benthic community dynamics along the North Coast of the Yucatan Peninsula, Mexico
Source: PLoS One. 2026 Mar 24;21(3):e0341611. doi: 10.1371/journal.pone.0341611 (PMC13012451; doi:10.1371/journal.pone.0341611)

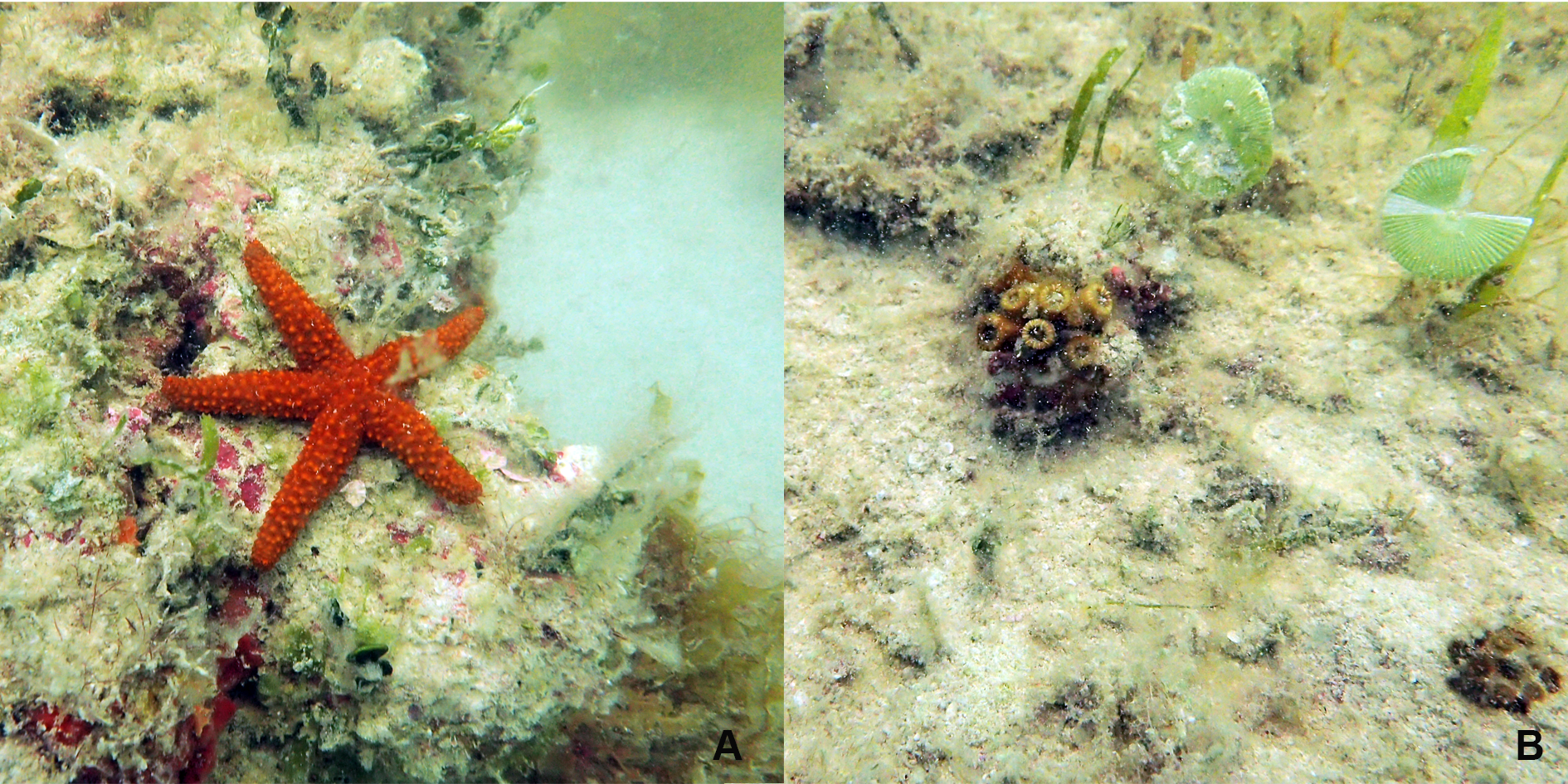

Supplement: S1 Fig — 1a) Substrate, LSAT and invertebrates, 1b) Substrate and Corals. (TIF) [file pone.0341611.s001.tif]

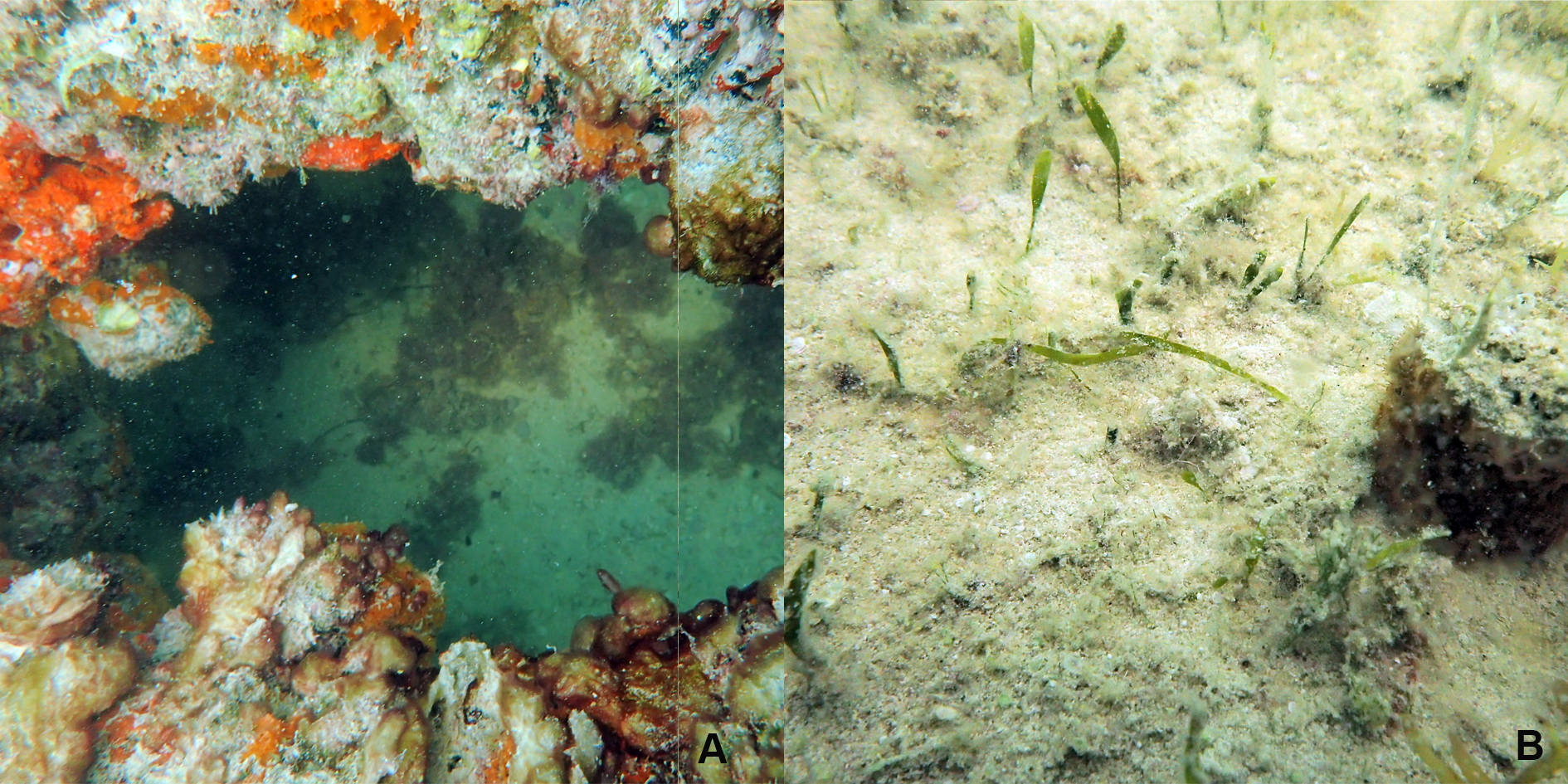

Supplement: S2 Fig — 2a) Photograph of the topography of SMR. 2b) Substrate LSAT, algae and sponge, 2b). (TIF) [file pone.0341611.s002.tif]

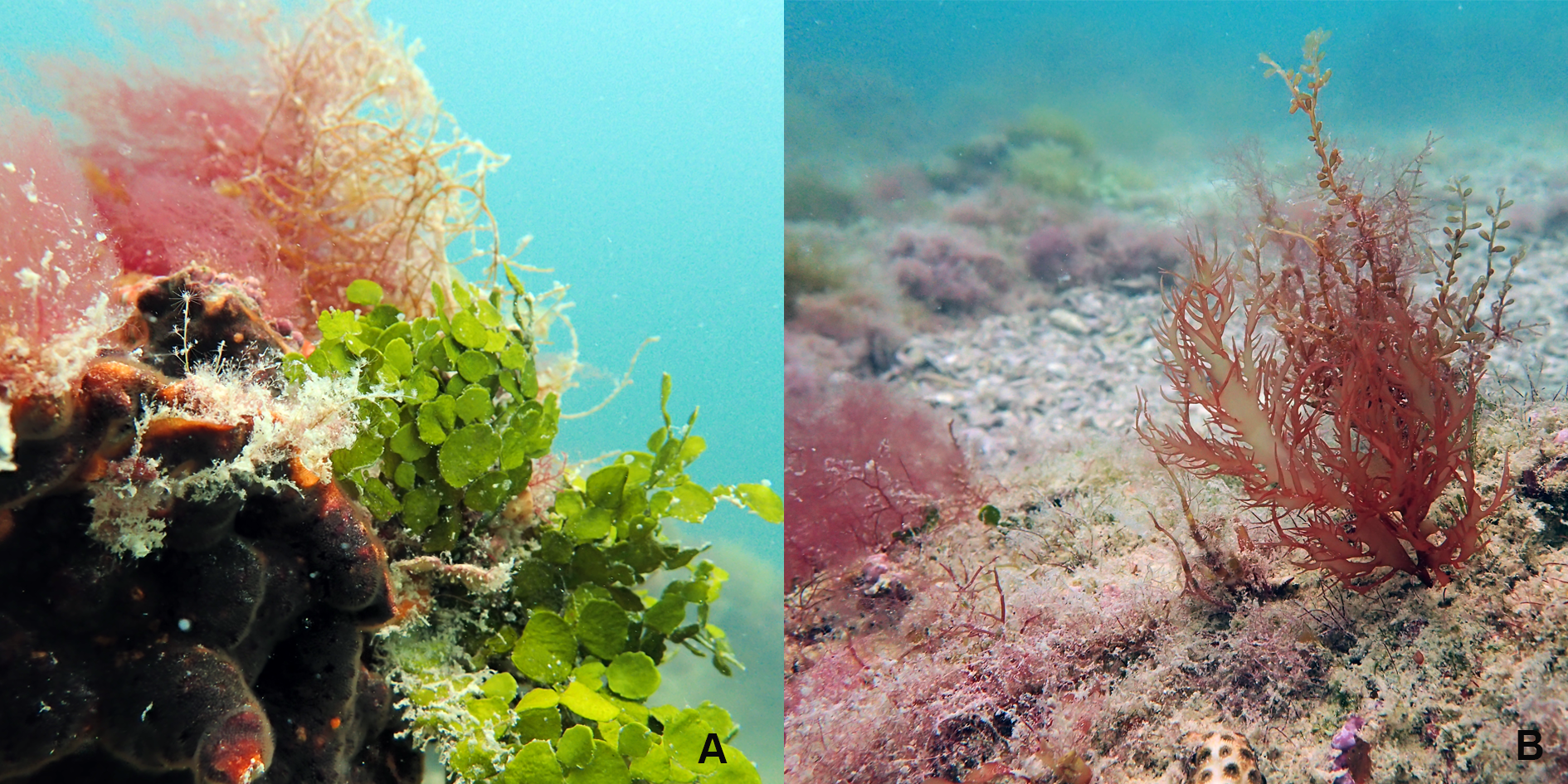

Supplement: S3 Fig — 3a) Photograph of the algae and sponge in Telchac rocky reef. 3b): Substrate, red algae and coral. (TIF) [file pone.0341611.s003.tif]

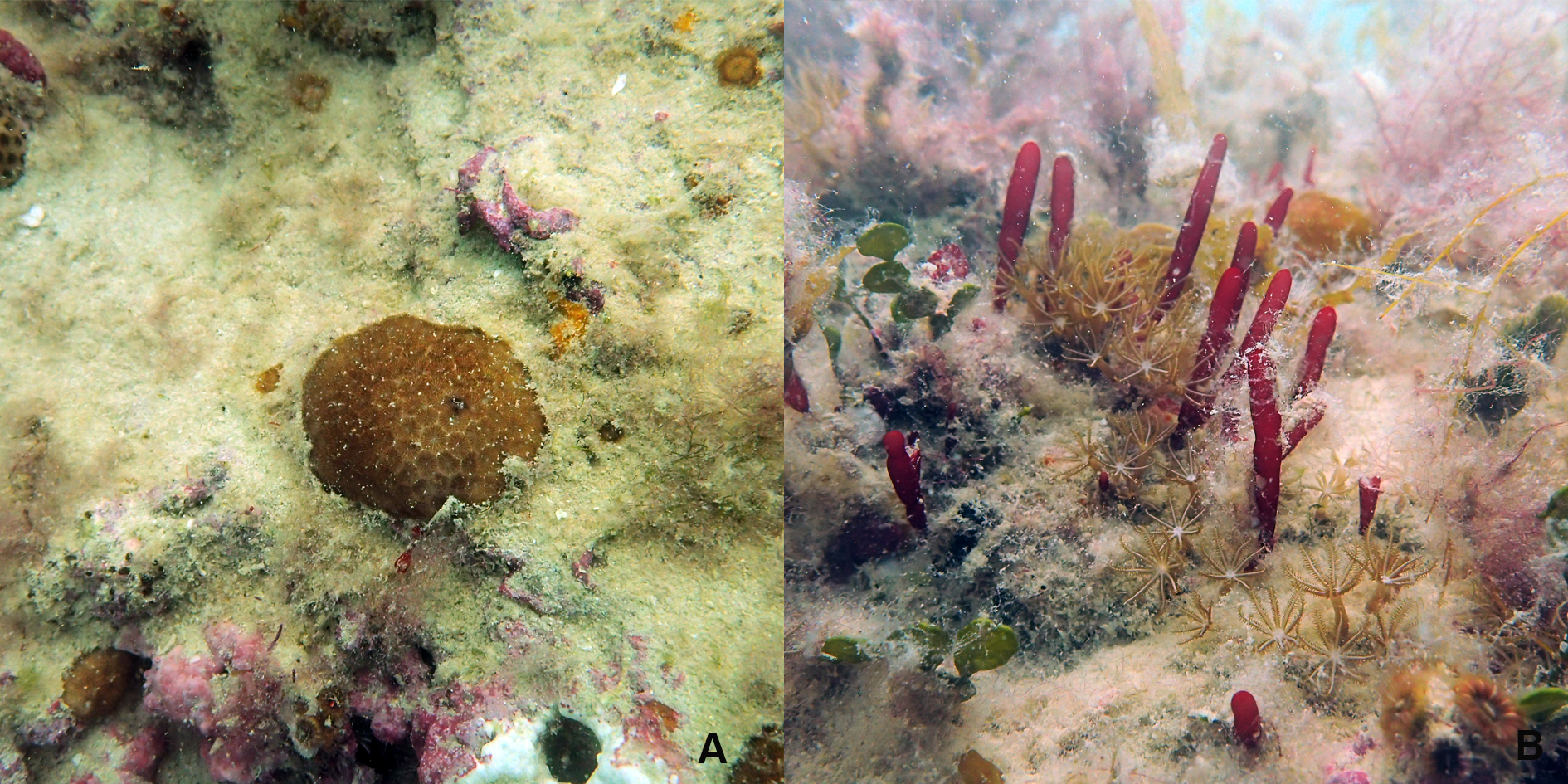

Supplement: S4 Fig — 4b) Red algae and invertebrates of Progreso rocky reef. (TIF) [file pone.0341611.s004.tif]

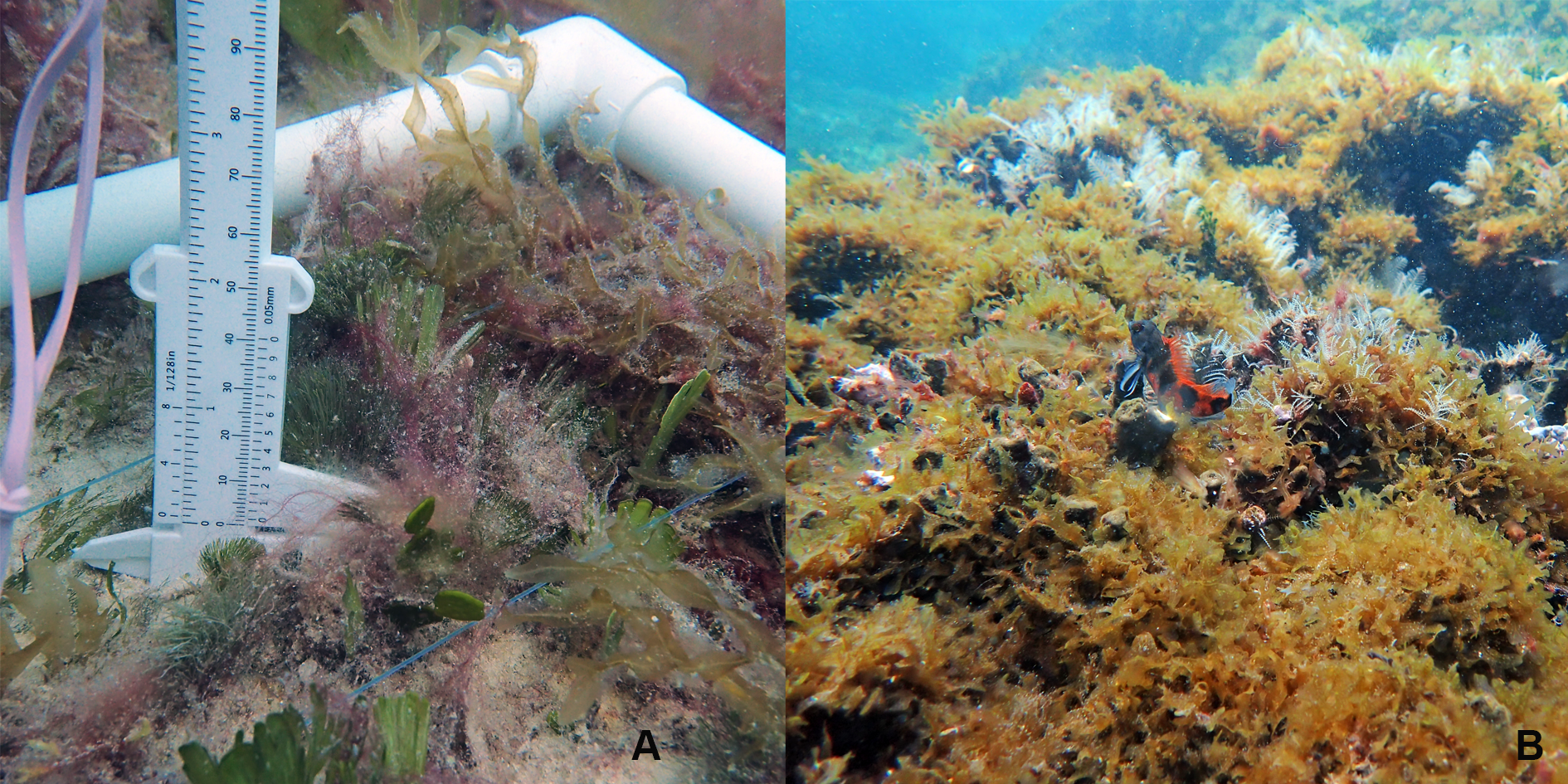

Supplement: S5 Fig — 5a) Photograph of Measurements of algae and substrate. 5b) Photograph of the bottom of Chicxulub rocky reef. (TIF) [file pone.0341611.s005.tif]
