# Supplementary material for: Unveiling hidden rocky reefs of the Mexican Atlantic coast: Topographic characterization and benthic community dynamics along the North Coast of the Yucatan Peninsula, Mexico
Source: PLoS One. 2026 Mar 24;21(3):e0341611. doi: 10.1371/journal.pone.0341611 (PMC13012451; doi:10.1371/journal.pone.0341611)
Supplement: Attachment — Submitted filename: response to reviewers.docx [file pone.0341611.s008.docx]

PONE-D-25-26194R1

"Unveiling Hidden Rocky Reefs of the Mexican Atlantic Coast: Topographic Characterization and Benthic Community Dynamics along the North Coast of the Yucatan Peninsula, Mexico,"

PLOS One


Reviewers' comments:

Please use the space provided to explain your answers to the questions above. You may also include additional comments for the author, including concerns about dual publication, research ethics, or publication ethics. (Please upload your review as an attachment if it exceeds 20,000 characters)

Reviewer #2: I thank the authors for their thorough responses to my comments. I think the ms will be suitable for publication following a minor revision. My suggestions are below and they should not be hard for the authors to address.
L27: “Rocky” should not be capitalised. **Done**
L63: I think these two sentences should be combined. Strange for the topic sentence to focus only on erosive degradation. Topic sentence should be on all threats to rocky reef habitats. **Done**
L234: I suggest rewording. An option – “Although not statistically significant (α = 0.05), the two-way ANOVA revealed a….”.**Done**
L244: Post-hoc comparisons should not be made as p > 0.05 for the interaction term. Instead talk about the trends in the data across seasons and reefs. **Done**
L252-L257: I would not say these associations are weak with these R squared values, especially in the field of ecology. Reword to ‘moderate’. **Done**
L262: remove the word “drivers”. That is causal language which is inappropriate given the statistical analyses used. I suggest “To identify the environmental variables that influence the abundance of macroalgae” or “To identify the environmental variables that are associated with the abundance of macroalgae”. **Done**
L337: Discussion should not have a “statistical analyses” subheading. Rather the results from the data analysis should be inform the ecological interpretation and discussion. **Done**
Figure 7 has not been updated. It should be presented as averages with standard errors.**DONE** I suggest bringing into the main text too. Also it should be referred to in the 2 way ANOVA results section. **Done in L242 in revised manuscript with track and in L237 in the manuscript with out track changes**

7. PLOS authors have the option to publish the peer review history of their article ([what does this mean?](https://journals.plos.org/plosone/s/editorial-and-peer-review-process#loc-peer-review-history)). If published, this will include your full peer review and any attached files.


If you choose “no”, your identity will remain anonymous but your review may still be made public.


**Do you want your identity to be public for this peer review?** For information about this choice, including consent withdrawal, please see our [Privacy Policy](https://www.plos.org/privacy-policy).

Reviewer #1: **Yes:**

Reviewer #2: No
